# Molecular Mechanisms of Vasculogenesis in Human Cancers: From Developmental Pathways to Tumor Vascular Remodeling and Therapeutic Targeting

**DOI:** 10.3390/biology15161362

**Published:** 2026-08-11

**Authors:** George-Jemal Gogitidze

**Affiliations:** School of Medicine and Healthcare Sciences, BAU International University, N237 Pridon Khalvashi Street, Batumi 6000, Georgia; jemal.gogitidze@bauinternational.edu.ge; Tel.: +995-592-58-02-02

**Keywords:** vasculogenesis, tumor vascular remodeling, endothelial progenitor cells, tumor microenvironment, endothelial-to-mesenchymal transition (EndMT), mechanotransduction

## Abstract

Vasculogenesis, the de novo formation of blood vessels from endothelial progenitor cells, is increasingly recognized as a critical contributor to tumor vascularization, complementing classical angiogenesis and promoting cancer progression, metastasis, therapeutic resistance, and disease recurrence. This narrative review aims to provide a comprehensive overview of the molecular mechanisms governing vasculogenesis across human malignancies and to discuss their translational relevance for targeted cancer therapy. This is a narrative, mechanistically oriented review rather than a systematic review or meta-analysis. The literature was searched in PubMed, Scopus, and Google Scholar, with the primary search covering publications from January 2010 to February 2026, while seminal earlier studies were included when they established fundamental concepts in vascular biology. Search terms included combinations of “vasculogenesis”, “tumor vasculogenesis”, “angiogenesis”, “endothelial progenitor cells”, “vascular remodeling”, “VEGF”, “HIF-1α”, “Notch”, “Wnt”, “PI3K/AKT”, “TGF-β”, “EndMT”, “mechanotransduction”, “non-coding RNAs”, “tumor microenvironment”, and “cancer”. Evidence was qualitatively appraised and synthesized according to mechanistic relevance and consistency across experimental and clinical studies. The reviewed literature highlights the complex interplay between developmental signaling pathways, endothelial plasticity, mechanical cues, inflammatory mediators, and non-coding RNAs in regulating tumor vasculogenesis. Collectively, these findings suggest that targeting vasculogenesis-related pathways may complement existing anti-angiogenic therapies and represent a promising strategy for improving precision oncology and overcoming treatment resistance.

## Simple Summary

Vasculogenesis, the de novo formation of blood vessels from endothelial progenitor cells, plays a fundamental role in embryonic development and contributes to tumor progression by establishing and remodeling the tumor vasculature. Beyond classical angiogenesis, tumor-associated vasculogenesis is increasingly recognized as a major driver of cancer growth, metastasis, therapeutic resistance, and relapse. This narrative review summarizes the molecular mechanisms that regulate vasculogenesis in human tumors, including VEGF, HIF-1α, Notch, Wnt/β-catenin, PI3K/AKT, TGF-β, and endothelial-to-mesenchymal transition (EndMT). We also discuss the impact of the tumor microenvironment, mechanotransduction, and non-coding RNAs on vascular remodeling, highlighting novel therapeutic strategies and future directions for targeting tumor vasculogenesis in precision oncology.

## 1. Introduction

The formation of an organized vascular network is essential for tissue development, homeostasis, and regeneration. Blood vessels ensure the delivery of oxygen and nutrients while facilitating immune surveillance and waste removal, thereby maintaining the physiological integrity of virtually every organ system [1]. During embryogenesis, the vascular system is established through vasculogenesis, a process involving the de novo differentiation of endothelial progenitor cells into primitive vascular structures. Subsequently, these vascular networks undergo expansion and remodeling through angiogenesis, arterialization, and vessel maturation to generate a functional circulatory system [2,3]. Although vasculogenesis was traditionally considered an embryonic event, accumulating evidence demonstrates that endothelial progenitor cells can also contribute to postnatal vascular repair and pathological neovascularization, particularly in malignant diseases [4].

The acquisition of an adequate blood supply represents one of the fundamental hallmarks of cancer. While angiogenesis has historically been regarded as the principal mechanism supporting tumor vascularization, recent studies have revealed that tumors exploit multiple complementary vascularization strategies, including vasculogenesis, vessel co-option, vascular mimicry, and intussusceptive angiogenesis [5,6]. Among these mechanisms, tumor vasculogenesis has emerged as an important contributor to neovascularization through the recruitment of bone marrow-derived endothelial progenitor cells and other vascular precursor populations to hypoxic tumor regions [7]. These cells integrate into developing vascular networks and interact with stromal, immune, and cancer cells, thereby promoting tumor growth, metastatic dissemination, and resistance to conventional anti-angiogenic therapies [8].

Tumor vasculogenesis is orchestrated by a highly complex molecular network involving developmental signaling pathways, hypoxia-responsive transcription factors, inflammatory mediators, and biomechanical stimuli. Key regulatory pathways include vascular endothelial growth factor (VEGF), hypoxia-inducible factor-1α (HIF-1α), phosphatidylinositol 3-kinase/protein kinase B (PI3K/AKT), Notch, Wnt/β-catenin, transforming growth factor-β (TGF-β), and stromal-derived factor-1 (SDF-1/CXCL12), which collectively regulate endothelial progenitor cell mobilization, migration, differentiation, and vascular remodeling [9,10,11,12]. In parallel, endothelial-to-mesenchymal transition (EndMT), extracellular matrix stiffness, mechanotransduction, and non-coding RNAs have emerged as critical modulators of vascular plasticity within the tumor microenvironment [13,14,15]. These discoveries have substantially expanded the understanding of vascular biology beyond classical angiogenesis. However, the clinical translation of these molecular insights remains incomplete, as many candidate therapeutic targets have yet to demonstrate consistent efficacy across different tumor types.

Despite considerable advances, several aspects of tumor vasculogenesis remain controversial. The precise contribution of endothelial progenitor cells to tumor vascularization varies among cancer types and experimental models, and the distinction between vasculogenesis and angiogenesis continues to be debated [16]. Furthermore, although VEGF-directed therapies have transformed the management of several malignancies, their long-term clinical benefits remain limited because tumors frequently activate compensatory vascularization mechanisms, including alternative developmental signaling pathways and vascular mimicry. In addition, the relative contribution of individual molecular pathways—including VEGF, Notch, Wnt/β-catenin, PI3K/AKT, and TGF-β—appears to vary substantially across tumor types, highlighting the absence of a universal model of tumor vasculogenesis [17]. These unresolved issues underscore the need for comparative mechanistic studies and clinically validated biomarkers capable of distinguishing vasculogenesis from other vascularization processes and identifying patients most likely to benefit from targeted therapies. Consequently, a more comprehensive understanding of the molecular mechanisms governing tumor vasculogenesis is required to improve therapeutic strategies and overcome treatment resistance.

This narrative review provides a comprehensive overview of the molecular mechanisms underlying vasculogenesis in human cancers, emphasizing developmental signaling pathways, endothelial progenitor cell biology, vascular remodeling, mechanotransduction, and the tumor microenvironment. Furthermore, the review discusses the role of vasculogenesis across major malignancies, and examines emerging biomarkers and therapeutic targets. Rather than providing only a descriptive overview, this review critically evaluates the current evidence, discusses areas of consensus and controversy, identifies major knowledge gaps, and highlights future research priorities required to translate mechanistic discoveries into clinically effective vasculogenesis-targeted therapies.

## 2. Methods

This narrative review was conducted through a comprehensive literature search aimed at summarizing current knowledge regarding the molecular mechanisms regulating vasculogenesis, endothelial progenitor cell biology, tumor-associated vascular remodeling, endothelial-to-mesenchymal transition (EndMT), and mechanotransduction in cancer. Relevant publications were identified through searches of PubMed/MEDLINE, Scopus, and Web of Science databases using combinations of keywords including “vasculogenesis,” “tumor vasculogenesis,” “endothelial progenitor cells,” “angiogenesis,” “VEGF,” “Notch,” “Wnt/β-catenin,” “TGF-β,” “BMP,” “PI3K/AKT,” “HIF-1α,” “EndMT,” “mechanotransduction,” and “vascular remodeling.” Original research articles, translational studies, and high-quality review articles published from database inception to June 2026 were considered based on their relevance to vascular development, pathological vascularization, and cancer progression. Studies lacking mechanistic information, non-peer-reviewed publications, conference abstracts without full manuscripts, and articles unrelated to vasculogenesis or tumor vascular biology were excluded. The selected literature was critically analyzed and synthesized according to major biological themes rather than quantitatively pooled. As a narrative review, this study has inherent limitations, including potential selection bias, heterogeneity among experimental models, variability in endothelial progenitor cell definitions, and difficulties distinguishing vasculogenesis from angiogenesis, vascular mimicry, and endothelial plasticity. These limitations highlight the need for future studies integrating advanced molecular and spatial approaches.

## 3. Physiological Vasculogenesis During Embryonic Development

Vasculogenesis is the process by which the first blood vessels are formed de novo from mesoderm-derived progenitor cells during embryogenesis and represents the earliest stage of vascular development. Unlike angiogenesis, which involves the sprouting and remodeling of pre-existing vessels, vasculogenesis establishes the primary vascular plexus that subsequently serves as the foundation for the mature circulatory system [18]. In mammals, this process begins shortly after gastrulation when mesodermal cells differentiate into hemangioblasts, bipotent progenitors capable of giving rise to both endothelial and hematopoietic lineages. Although the existence of a common hemangioblast remains debated, substantial experimental evidence supports the presence of endothelial precursor populations that contribute to early vascular formation [19].

The specification of endothelial cells is tightly regulated by a coordinated network of transcription factors and signaling pathways. **Fibroblast growth factor (FGF)** and **bone morphogenetic proteins (BMPs)** initiate mesodermal differentiation, whereas **vascular endothelial growth factor A (VEGF-A)** acts as the principal driver of endothelial lineage commitment through activation of **VEGF receptor-2 (VEGFR-2/KDR/Flk-1)**. Binding of VEGF-A to VEGFR-2 activates intracellular signaling cascades, including the **PI3K/AKT**, **MAPK/ERK**, and **PLCγ** pathways, promoting endothelial cell survival, proliferation, migration, and differentiation [20]. The transcription factors **ETV2**, **SCL/TAL1**, **GATA2**, and **ERG** further regulate endothelial gene expression and establish endothelial cell identity, while suppression of alternative mesodermal fates ensures vascular specification [21].

As endothelial progenitor cells differentiate, they migrate and assemble into primitive vascular cords through coordinated cell–cell adhesion mediated by **VE-cadherin**, integrins, and extracellular matrix proteins such as fibronectin and laminin. These solid endothelial cords subsequently undergo lumen formation through intracellular vacuole fusion, polarization of endothelial cells, and cytoskeletal remodeling, ultimately generating a functional vascular lumen capable of supporting blood flow [22]. Simultaneously, primitive endothelial cells secrete extracellular matrix components that stabilize the nascent vascular network.

The maturation and remodeling of embryonic vessels depend on continuous communication between endothelial cells and their surrounding microenvironment. **Notch signaling** regulates arterial specification through interactions between the ligand **DLL4** and Notch receptors, whereas **EphrinB2** and **EphB4** establish arterial and venous identity, respectively. Recruitment of pericytes and vascular smooth muscle cells is primarily mediated by **platelet-derived growth factor-B (PDGF-B)** and **transforming growth factor-β (TGF-β)**, promoting vessel stabilization and preventing regression [23]. In addition, blood flow-generated shear stress activates mechanotransduction pathways involving **KLF2**, **YAP/TAZ**, and endothelial nitric oxide synthase (eNOS), which further regulate endothelial differentiation, vascular remodeling, and functional maturation [24]. See Table 1 for the summary of all molecular mechanisms involved in embryonic vasculogenesis.

Despite significant advances in elucidating the molecular mechanisms of embryonic vasculogenesis, several fundamental questions regarding its regulation, cellular origins, and translational relevance remain unresolved. Although developmental signaling pathways such as VEGF, Notch, BMP, and FGF have been extensively characterized in experimental models, their precise temporal hierarchy, context-dependent interactions, and relative contributions during human vascular development remain incompletely understood. Furthermore, the long-standing concept of the hemangioblast as a common progenitor of endothelial and hematopoietic cells continues to be debated, with emerging lineage-tracing studies suggesting that endothelial progenitor populations may arise through multiple developmental routes rather than from a single bipotent precursor. Another unresolved issue concerns the extent to which molecular mechanisms identified during embryogenesis are faithfully recapitulated in pathological conditions such as cancer. While tumors frequently reactivate embryonic signaling programs, the tumor microenvironment introduces additional influences—including chronic hypoxia, inflammation, immune cell infiltration, and extracellular matrix remodeling—that may fundamentally alter the regulation of vasculogenesis. Consequently, direct extrapolation of developmental biology findings to tumor-associated vascularization should be approached with caution. Future studies integrating single-cell transcriptomics, spatial omics, advanced lineage-tracing technologies, and functional validation in human tissues will be essential to clarify the cellular origins, regulatory interactions, and temporal dynamics of vasculogenesis in both physiological development and human malignancies. Such approaches may also help determine which developmental mechanisms are truly conserved and therefore represent realistic therapeutic targets in cancer.

**Figure 1:**
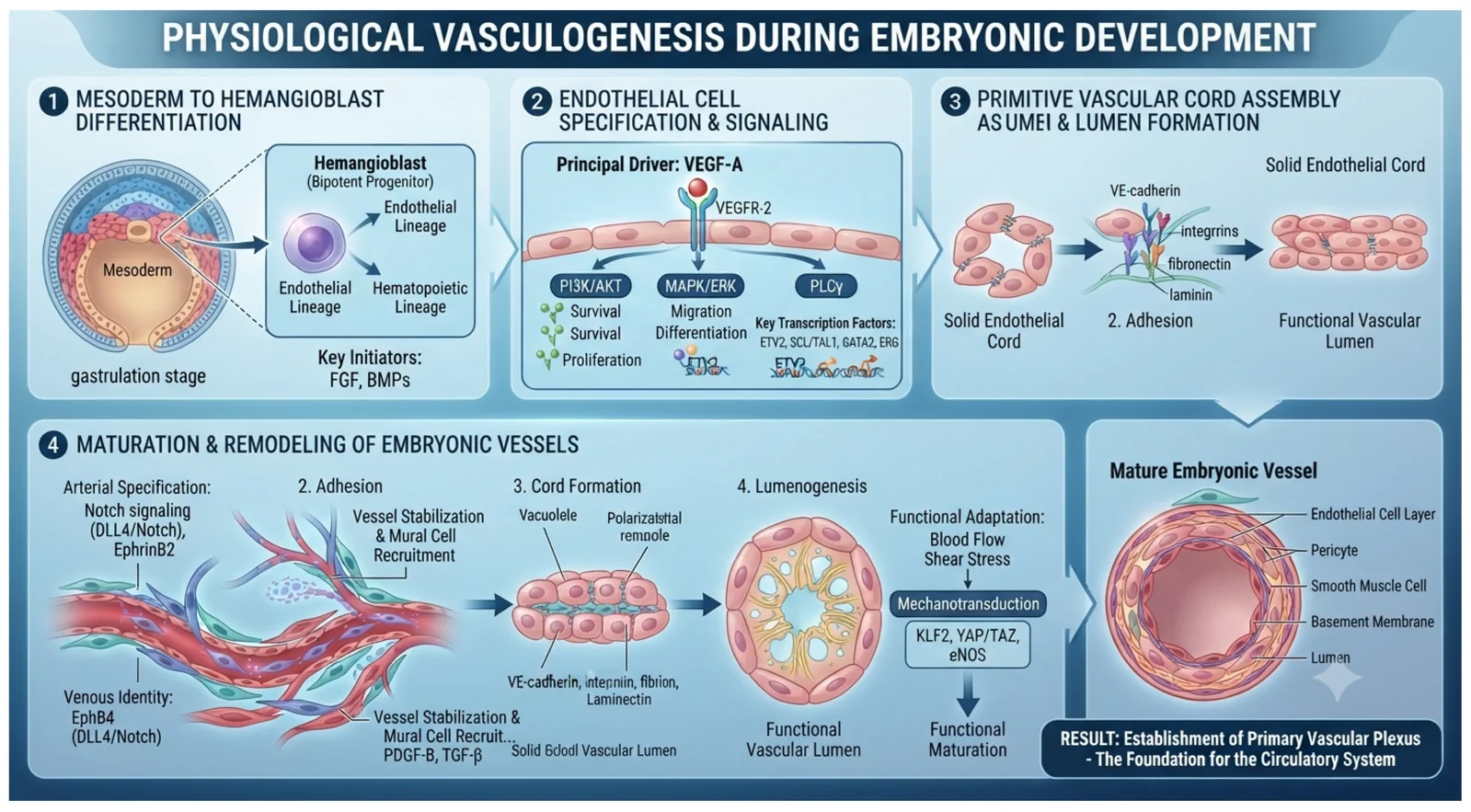
Molecular and Cellular Mechanisms Regulating Physiological Vasculogenesis During Embryonic Development. Physiological vasculogenesis begins when mesodermal cells differentiate into hemangioblasts, which then commit to the endothelial lineage via VEGF-A and VEGFR-2 signaling. These specified endothelial cells assemble into primitive vascular cords, undergo lumen formation and polarization, and subsequently mature and stabilize through arterial–venous specification, Mural Cell Recruitment, and mechanotransduction.

Collectively, embryonic vasculogenesis represents a highly coordinated developmental program integrating genetic, molecular, and biomechanical signals to establish the primary vascular network. Many developmental signaling pathways appear to be reactivated during tumor progression. However, accumulating evidence suggests that their regulation within the tumor microenvironment is substantially more complex than during embryogenesis, owing to the influence of hypoxia, inflammatory signaling, stromal interactions, and tumor heterogeneity. Understanding these context-dependent differences remains a major challenge for translating developmental vascular biology into effective anticancer therapies [25]. See Figure 1 to understand the main mechanisms involved in physiological vasculogenesis during embryonic development.

## 4. Molecular Regulation of Vasculogenesis (VEGF, Notch, Wnt, BMP, TGF-β, PI3K/AKT, HIF-1α)

Vasculogenesis is regulated by an intricate network of molecular pathways that coordinate endothelial progenitor cell specification, proliferation, migration, differentiation, and vascular maturation. These signaling cascades integrate developmental programs, metabolic adaptation, extracellular matrix interactions, and environmental stimuli to establish functional vascular networks. Importantly, many of the pathways involved in physiological vasculogenesis are reactivated during pathological conditions, particularly cancer, where they contribute to tumor vascularization, vascular remodeling, and resistance to anti-angiogenic therapies [26].

However, despite extensive characterization of these signaling pathways, the precise contribution of individual molecular regulators to adult vasculogenesis remains controversial. Most mechanistic insights originate from embryonic development models or experimental systems, and their relevance to human pathological conditions, particularly cancer, remains incompletely defined. A major unresolved question is whether tumor-associated vasculogenesis represents true endothelial neogenesis or rather a spectrum of endothelial remodeling processes involving endothelial plasticity, vessel co-option, and recruitment of progenitor-like cells.

### 4.1. Vascular Endothelial Growth Factor (VEGF) Signaling

**Vascular Endothelial Growth Factor (VEGF) signaling** represents the primary molecular driver of endothelial development and vasculogenesis. VEGF-A is the dominant ligand responsible for endothelial lineage commitment and vascular growth through interaction with VEGF receptor-2 (VEGFR-2/KDR/Flk-1) expressed on endothelial progenitor cells and immature endothelial cells. VEGFR-2 activation initiates multiple intracellular cascades, including **phosphatidylinositol 3-kinase/protein kinase B (PI3K/AKT)**, **RAS/mitogen-activated protein kinase (MAPK/ERK)**, and **phospholipase C-γ (PLCγ)** pathways. These mechanisms promote endothelial cell survival, proliferation, migration, cytoskeletal remodeling, and differentiation. Activation of PI3K/AKT signaling additionally enhances endothelial nitric oxide synthase (eNOS) activity and nitric oxide production, facilitating endothelial migration and vascular maturation [27]. The balance of VEGF signaling intensity is essential, as excessive activation may produce abnormal vascular structures, whereas insufficient signaling results in impaired vascular development.

Despite its central role, VEGF signaling alone cannot fully explain the complexity of vascular formation in tumors. Clinical experience with VEGF-targeted therapies has demonstrated that inhibition of VEGF frequently produces transient responses followed by adaptive resistance, suggesting the existence of VEGF-independent mechanisms of vascular regeneration. Alternative pathways, including fibroblast growth factor (FGF), angiopoietin/Tie signaling, platelet-derived growth factor (PDGF), and inflammatory cytokine networks, may compensate for VEGF blockade. Furthermore, VEGF expression levels do not consistently predict therapeutic response, highlighting the need for more reliable biomarkers that reflect the functional state of tumor vascular regulation rather than single-factor expression.

### 4.2. Notch Signaling

**Notch signaling** is a fundamental regulator of endothelial specification, arterial–venous differentiation, and vascular patterning. The interaction between Notch receptors, particularly Notch1, and endothelial ligands such as Delta-like ligand 4 (DLL4), determines endothelial cell fate and controls the balance between endothelial sprouting and vessel maturation. Notch activation induces arterial-specific transcriptional programs mediated by HEY1 and HEY2 while limiting excessive endothelial proliferation and maintaining vascular organization. Importantly, VEGF and Notch pathways exhibit bidirectional regulation, with VEGF promoting endothelial activation and Notch controlling subsequent vascular stabilization and specialization [28].

However, the therapeutic targeting of Notch signaling remains challenging because its effects are highly context-dependent. Although DLL4–Notch activation suppresses excessive endothelial sprouting and promotes vessel maturation in developmental models, inhibition of this pathway can paradoxically induce excessive but dysfunctional angiogenesis. Moreover, whether modulation of Notch signaling can selectively inhibit pathological vasculogenesis without disrupting physiological vascular maintenance remains unresolved.

### 4.3. The Wnt/β-Catenin Pathway

**The Wnt/β-catenin pathway** contributes to endothelial lineage commitment and maintenance of vascular progenitor populations. Activation of canonical Wnt signaling prevents β-catenin degradation, allowing its nuclear translocation and regulation of endothelial-specific transcriptional programs. Wnt signaling cooperates with VEGF and Notch pathways to regulate endothelial differentiation, proliferation, and vascular network formation during embryonic development [29].

Nevertheless, the precise role of Wnt signaling in adult vasculogenesis remains debated. While canonical Wnt activation supports endothelial progenitor specification during development, persistent Wnt pathway activation in cancer may promote vascular abnormalities through interactions with tumor cells, immune cells, and extracellular matrix components. The difficulty in distinguishing developmental vascular programs from tumor-specific reactivation represents a major challenge for therapeutic targeting of this pathway.

### 4.4. Bone Morphogenetic Protein (BMP) Signaling

**Bone Morphogenetic Protein (BMP) signaling**, belonging to the transforming growth factor-β (TGF-β) superfamily, plays a critical role during early mesoderm specification and hemangioblast formation. BMP4-mediated activation of SMAD-dependent transcription promotes endothelial progenitor generation and vascular lineage differentiation. Alterations in BMP signaling can disrupt vascular development and contribute to congenital vascular abnormalities [30].

Despite these observations, the contribution of BMP signaling to pathological vasculogenesis remains insufficiently understood. BMP effects appear highly dependent on ligand type, receptor expression pattern, and cellular context. For example, BMP-mediated endothelial differentiation during development may contrast with its potential role in tumor progression, where BMP signaling can influence stromal remodeling, immune regulation, and metastatic behavior. Further studies are required to define whether BMP pathways represent therapeutic targets or merely markers of vascular remodeling.

### 4.5. TGF-β Signaling

**TGF-β signaling** exerts complex, context-dependent effects on vascular development. Through activation of SMAD2/3-dependent pathways, TGF-β regulates endothelial differentiation, extracellular matrix remodeling, vascular stabilization, and communication between endothelial cells and mural cells. Furthermore, TGF-β is a key inducer of endothelial-to-mesenchymal transition (EndMT), a process involved in physiological vascular remodeling and pathological vascular transformation within tumors [31].

The dual nature of TGF-β signaling represents a major unresolved issue. Although TGF-β contributes to vascular stabilization and tissue repair under physiological conditions, excessive activation may promote fibrosis, endothelial dysfunction, and tumor-associated vascular abnormalities. In addition, the quantitative contribution of EndMT-derived cells to functional tumor vasculature remains controversial, with some studies suggesting substantial involvement, while others indicate a limited contribution compared with endothelial cell plasticity and perivascular remodeling.

### 4.6. Hypoxia-Inducible Factor-1α (HIF-1α)

**Hypoxia-inducible factor-1α (HIF-1α)** functions as a central oxygen-sensitive regulator of vasculogenesis. Under hypoxic conditions, HIF-1α escapes degradation, accumulates in the nucleus, and activates transcription of multiple pro-vasculogenic genes, including VEGF-A, stromal-derived factor-1 (SDF-1/CXCL12), and angiopoietins. This pathway promotes endothelial progenitor cell mobilization, migration toward hypoxic tissues, and adaptation of vascular networks to metabolic demands [32]. In tumors, persistent hypoxia-mediated HIF-1α activation represents a major mechanism driving pathological vasculogenesis and vascular remodeling.

However, hypoxia-induced HIF-1α activation does not uniformly result in productive vascularization. Chronic tumor hypoxia may generate abnormal, immature, and inefficient vascular networks characterized by increased permeability and poor perfusion. Therefore, an important unanswered question is whether therapeutic strategies should suppress hypoxia-driven vascular responses or instead promote vascular normalization to improve oxygen delivery and enhance treatment efficacy.

Collectively, VEGF, Notch, Wnt, BMP, TGF-β, PI3K/AKT, and HIF-1α pathways form an interconnected regulatory network controlling vascular development. Understanding these mechanisms provides critical insights into the transition from physiological vasculogenesis to pathological tumor-associated vascular formation and identifies potential therapeutic targets for cancer treatment [33].

### 4.7. Integration and Cancer-Specific Divergence of Vasculogenic Signaling Pathways

Although VEGF, Notch, Wnt, BMP, TGF-β, PI3K/AKT, and HIF-1α pathways are frequently described as individual regulators of vasculogenesis, accumulating evidence indicates that tumor vascular formation is governed by complex pathway interactions rather than isolated signaling events. HIF-1α represents an upstream environmental regulator that links hypoxia to vasculogenic activation by inducing VEGF, CXCL12/CXCR4, angiopoietins, and other mediators responsible for endothelial progenitor cell recruitment and vascular adaptation [11,32]. VEGF primarily initiates endothelial activation, proliferation, and migration; however, the subsequent organization and maturation of vascular structures depend strongly on Notch-mediated endothelial specification and stabilization [27,28]. Therefore, excessive VEGF activity in the absence of appropriate Notch signaling may generate immature and dysfunctional vessels, a characteristic feature of many aggressive tumors.

Furthermore, PI3K/AKT signaling functions as a major intracellular convergence pathway downstream of multiple receptors, including VEGFR, growth factor receptors, and inflammatory mediators, promoting endothelial survival, metabolic adaptation, and resistance to vascular-targeted therapies [27,33]. In contrast, Wnt and BMP pathways appear to have more context-dependent roles, being essential for endothelial progenitor specification during development but potentially contributing to tumor vascular plasticity when aberrantly reactivated. TGF-β represents another pathway with dual functions, supporting vascular stabilization under physiological conditions while promoting endothelial-to-mesenchymal transition and stromal remodeling in cancer.

### 4.8. Current Controversies and Unresolved Questions in Molecular Regulation of Vasculogenesis

Although major molecular pathways regulating vasculogenesis have been extensively characterized, several fundamental questions remain unresolved. First, the relative contribution of vasculogenesis compared with angiogenesis in human tumors remains controversial. While experimental models demonstrate incorporation of progenitor-derived endothelial cells into tumor vessels, lineage-tracing studies have questioned the extent to which circulating endothelial progenitor cells contribute directly to functional vasculature.

Second, the definition and identification of endothelial progenitor cells remain inconsistent. Different studies use heterogeneous surface markers, including CD34, CD133, VEGFR-2, and other endothelial-associated markers, resulting in substantial variability in reported biological functions. This lack of standardized characterization limits comparison between studies and complicates clinical translation.

Third, most current knowledge is derived from simplified experimental models that do not fully reproduce the complexity of human tumors, where endothelial cells interact with immune populations, cancer-associated fibroblasts, extracellular matrix components, and metabolic gradients. Emerging technologies, including single-cell transcriptomics, spatial molecular profiling, and advanced imaging approaches, may provide more precise insights into the cellular origin and functional significance of tumor vasculogenesis.

Therefore, future research should focus not only on identifying individual molecular regulators but also on understanding how integrated signaling networks coordinate vascular plasticity, therapeutic resistance, and tumor evolution.

## 5. Endothelial Progenitor Cells and Vasculogenesis

Endothelial progenitor cells (EPCs) are specialized precursor cells that play a pivotal role in de novo blood vessel formation and vascular regeneration. First identified by Asahara et al. in 1997, EPCs challenged the long-standing concept that postnatal vascular growth occurs exclusively through angiogenesis by demonstrating that circulating progenitor cells can differentiate into mature endothelial cells and contribute to vasculogenesis [34]. During embryonic development, endothelial progenitors arise primarily from mesoderm-derived hemangioblasts and angioblasts, whereas in adults, EPCs are predominantly mobilized from the bone marrow into the peripheral circulation in response to tissue injury, ischemia, hypoxia, or inflammatory stimuli [35].

EPC mobilization and recruitment are tightly regulated by multiple molecular pathways, including vascular endothelial growth factor (VEGF), stromal-derived factor-1 (SDF-1/CXCL12), hypoxia-inducible factor-1α (HIF-1α), granulocyte colony-stimulating factor (G-CSF), angiopoietins, and nitric oxide signaling [36]. Once recruited to sites of vascular remodeling, EPCs adhere to the extracellular matrix, proliferate, migrate, and differentiate into endothelial cells, thereby contributing to the formation and stabilization of newly developing vascular networks. Beyond their direct incorporation into blood vessels, EPCs exert potent paracrine effects by secreting proangiogenic cytokines, growth factors, extracellular vesicles, and microRNAs that stimulate endothelial survival, vascular remodeling, and tissue repair [37].

In cancer, tumor-derived hypoxia and inflammatory mediators promote continuous EPC mobilization and homing to the tumor microenvironment, where they participate in pathological vasculogenesis and support tumor growth, metastatic dissemination, and therapeutic resistance. Consequently, EPCs have emerged as promising biomarkers of tumor progression and attractive targets for novel anti-vascular therapeutic strategies [38].

Nevertheless, the biological identity and functional importance of EPCs remain subjects of considerable debate. Subsequent studies have demonstrated that many circulating cells initially classified as EPCs represent hematopoietic populations with proangiogenic paracrine activity rather than true endothelial precursors. The lack of universally accepted EPC markers has resulted in conflicting conclusions regarding their contribution to vessel formation. Consequently, future studies employing lineage tracing and single-cell approaches are required to determine whether EPCs primarily function through direct endothelial incorporation or indirect regulation of vascular remodeling.

A major challenge in EPC research is the absence of standardized phenotypic criteria for their identification. Traditionally, EPCs have been defined using combinations of surface markers, including CD34, CD133, VEGFR-2 (KDR), and other endothelial-associated antigens; however, these markers are not exclusively expressed by EPCs and may overlap with hematopoietic progenitor cells, monocyte-derived populations, and immature endothelial cells. Differences in isolation strategies, culture conditions, and functional assays have therefore resulted in the identification of heterogeneous cell populations that may not represent a single biological entity. Moreover, findings obtained from experimental models, particularly animal lineage-tracing studies, cannot always be directly extrapolated to human tumors, where circulating progenitor-like cells are influenced by complex interactions with immune cells, stromal components, and the extracellular matrix. Thus, the precise contribution of EPCs to human tumor vasculogenesis remains difficult to quantify, and advanced approaches such as single-cell transcriptomics, spatial profiling, and standardized functional assays are required to distinguish true endothelial progenitor activity from broader mechanisms of vascular plasticity and remodeling.

## 6. Tumor Vasculogenesis Versus Angiogenesis

Although the terms vasculogenesis and angiogenesis are often used interchangeably, they represent distinct biological processes that contribute to tumor vascularization. Angiogenesis refers to the formation of new blood vessels through the sprouting, branching, and remodeling of pre-existing vascular networks, whereas vasculogenesis involves the de novo generation of blood vessels through the recruitment, migration, and differentiation of endothelial progenitor cells (EPCs), primarily derived from the bone marrow [39]. Both mechanisms are essential for supplying oxygen and nutrients to rapidly growing tumors; however, their relative contribution varies according to tumor type, stage, and microenvironmental conditions.

Importantly, the distinction between vasculogenesis and angiogenesis in tumors is increasingly recognized as less clear-cut than previously assumed. Tumor vascularization represents a dynamic continuum involving endothelial sprouting, vessel co-option, endothelial plasticity, vasculogenic mimicry, and recruitment of progenitor-like cells. The extent to which classical vasculogenesis contributes to human tumor progression remains controversial, partly due to limitations in experimental models and difficulties in accurately identifying progenitor-derived endothelial cells within complex tumor tissues.

Tumor angiogenesis is predominantly driven by local endothelial cell activation in response to proangiogenic factors, particularly vascular endothelial growth factor (VEGF), fibroblast growth factors (FGFs), platelet-derived growth factor (PDGF), and angiopoietins. In contrast, tumor vasculogenesis is initiated by hypoxia-induced stabilization of hypoxia-inducible factor-1α (HIF-1α), which stimulates the expression of VEGF, stromal-derived factor-1 (SDF-1/CXCL12), and other chemotactic molecules that mobilize EPCs from the bone marrow to the tumor microenvironment [40]. After homing to hypoxic regions, EPCs differentiate into endothelial cells and integrate into developing vascular networks while also secreting paracrine mediators that enhance vascular remodeling and endothelial survival.

Importantly, accumulating evidence indicates that vasculogenesis contributes to resistance against anti-angiogenic therapies by providing an alternative mechanism of tumor revascularization following inhibition of local endothelial sprouting. Consequently, simultaneous targeting of both angiogenic and vasculogenic pathways has emerged as a promising therapeutic strategy to improve vascular normalization and overcome treatment resistance in human cancers [41]. See Table 2 for the main differences between vasculogenesis and angiogenesis.

## 7. Vasculogenesis in Major Cancers

Tumor vasculogenesis contributes to the progression of numerous malignancies by promoting the recruitment of endothelial progenitor cells (EPCs), vascular remodeling, and the establishment of an adequate blood supply for tumor growth. Although the relative contribution of vasculogenesis differs among cancer types, activation of the hypoxia-inducible factor-1α (HIF-1α)/vascular endothelial growth factor (VEGF) axis remains a common mechanism underlying pathological neovascularization [42].

However, the biological significance of tumor vasculogenesis remains highly heterogeneous and appears to depend on tumor origin, genetic background, vascular architecture, and the composition of the tumor microenvironment. Although EPC recruitment and incorporation have been demonstrated in several experimental models, the extent to which vasculogenesis represents a dominant mechanism of vascular formation in human malignancies remains controversial. In many tumors, vasculogenesis likely functions as a complementary process interacting with classical angiogenesis, vessel co-option, and vasculogenic mimicry rather than acting as an independent vascularization mechanism.

Across malignancies, pathogenic vasculogenesis manifests through several recurring biological patterns rather than a single universal mechanism. These include hypoxia-driven recruitment of endothelial progenitor cells, activation of VEGF/HIF-1α-dependent vascular remodeling, interaction with inflammatory and stromal components of the tumor microenvironment, and acquisition of vascular plasticity through mechanisms such as endothelial transdifferentiation and vascular mimicry. However, the relative contribution of each mechanism varies among tumor types, reflecting differences in genetic alterations, tissue origin, and microenvironmental conditions.

In **breast cancer**, hypoxia-induced mobilization of EPCs and activation of VEGF, CXCL12/CXCR4, and PI3K/AKT signaling enhance vascular remodeling, facilitating tumor progression, metastatic dissemination, and resistance to anti-angiogenic therapies [43]. **Lung cancer**, particularly non-small cell lung cancer, demonstrates increased EPC recruitment driven by HIF-1α, VEGF, and inflammatory cytokines, contributing to rapid tumor vascularization and poor clinical outcomes [44]. In **colorectal cancer**, vasculogenesis promotes liver metastasis through coordinated activation of VEGF, Wnt/β-catenin, and TGF-β signaling, while EPC infiltration correlates with advanced tumor stage and unfavorable prognosis [45].

Nevertheless, the clinical relevance of EPC-mediated vasculogenesis in solid tumors remains uncertain. Studies investigating EPC abundance as a prognostic marker have produced inconsistent results, partly because different studies use variable definitions and surface markers for EPC identification. For example, CD34, CD133, VEGFR-2, and other endothelial-associated markers may identify overlapping but biologically distinct cell populations, complicating comparison between tumor types. Therefore, whether EPC levels directly reflect vasculogenic activity or simply represent systemic responses to tumor-associated inflammation remains unresolved.

Among primary brain tumors, **glioblastoma** exhibits one of the highest levels of tumor vasculogenesis. Severe intratumoral hypoxia stimulates continuous EPC mobilization and vascular remodeling, resulting in highly abnormal vascular networks that contribute to treatment resistance and recurrence [46]. In **hepatocellular carcinoma**, chronic inflammation, cirrhosis, and hypoxia activate VEGF, angiopoietins, and HIF-1α signaling, promoting vasculogenesis that supports tumor growth and intrahepatic spread [47]. Similarly, **renal cell carcinoma**, characterized by frequent von Hippel–Lindau (VHL) gene alterations, exhibits constitutive HIF activation and excessive VEGF production, making vasculogenesis a major determinant of its highly vascular phenotype [48].

Despite strong experimental evidence supporting vasculogenic mechanisms in highly vascular tumors such as glioblastoma and renal cell carcinoma, important limitations remain. Glioblastoma-associated vascular remodeling is particularly complex because tumor vessels may arise through multiple mechanisms, including angiogenesis, vascular mimicry, endothelial transdifferentiation, and recruitment of progenitor-like cells. Similarly, although VHL loss provides a strong mechanistic link between hypoxia signaling and VEGF activation in renal cell carcinoma, VEGF dependency varies among patients, contributing to heterogeneous responses to antiangiogenic therapies.

In **ovarian cancer**, EPC recruitment is stimulated by hypoxia and inflammatory cytokines within the peritoneal microenvironment, enhancing tumor vascularization and peritoneal dissemination [49]. **Cervical cancer** demonstrates progressive activation of vasculogenic pathways during malignant transformation, with persistent hypoxia and HPV-associated molecular alterations increasing VEGF expression and endothelial progenitor cell recruitment [50]. Finally, **melanoma** displays remarkable vascular plasticity, utilizing both vasculogenesis and alternative vascularization mechanisms, including vascular mimicry, to sustain metastatic growth and evade anti-vascular therapies [51].

These observations highlight an important unresolved question: whether targeting vasculogenesis alone can provide meaningful therapeutic benefit. Tumors frequently demonstrate vascular plasticity, allowing rapid adaptation following inhibition of a single vascular pathway. In melanoma and other highly adaptable malignancies, alternative mechanisms such as vascular mimicry, vessel co-option, and endothelial cell plasticity may compensate for impaired vasculogenesis. Therefore, successful therapeutic strategies may require simultaneous targeting of multiple vascular remodeling processes rather than inhibition of individual signaling pathways.

Collectively, current evidence indicates that tumor vasculogenesis represents a context-dependent and highly dynamic process rather than a universal mechanism of tumor vascularization. Although HIF-1α, VEGF, CXCL12/CXCR4, and inflammatory signaling pathways are frequently involved, their relative importance varies substantially between malignancies. A major challenge in the field is the absence of standardized approaches to quantify vasculogenesis in human tumors and distinguish it from angiogenesis, vascular mimicry, and endothelial plasticity. Future studies integrating lineage tracing, spatial transcriptomics, single-cell sequencing, and advanced imaging approaches are required to define the true contribution of vasculogenesis to cancer progression and identify patients who may benefit from vasculogenesis-targeted therapies [52].

## 8. Endothelial-to-Mesenchymal Transition (EndMT)

Endothelial-to-mesenchymal transition (EndMT) is a dynamic biological process in which endothelial cells progressively lose their characteristic phenotype and acquire mesenchymal, fibroblast-like properties. During EndMT, endothelial markers, including vascular endothelial cadherin (VE-cadherin), platelet endothelial cell adhesion molecule-1 (CD31), and endothelial nitric oxide synthase (eNOS), are downregulated, whereas mesenchymal markers such as α-smooth muscle actin (α-SMA), N-cadherin, vimentin, fibronectin, and fibroblast-specific protein-1 (FSP-1) become upregulated [53]. This phenotypic transition is regulated by several interconnected signaling pathways, particularly transforming growth factor-β (TGF-β), Notch, Wnt/β-catenin, hypoxia-inducible factor-1α (HIF-1α), nuclear factor-κB (NF-κB), and phosphatidylinositol 3-kinase/protein kinase B (PI3K/AKT), which activate transcription factors including Snail, Slug, Twist, and ZEB1/2 [54].

Physiologically, EndMT contributes to embryonic cardiovascular development and heart valve formation; however, persistent activation in adults is associated with fibrosis, chronic inflammation, and cancer progression [55]. Within the tumor microenvironment, hypoxia, inflammatory cytokines, and extracellular matrix remodeling promote EndMT, generating cancer-associated fibroblasts and other stromal cells that support tumor growth, angiogenesis, vasculogenesis, immune evasion, and metastatic dissemination [56]. Moreover, EndMT contributes to resistance against anti-angiogenic therapies by increasing endothelial plasticity and facilitating vascular remodeling despite therapeutic inhibition of VEGF signaling [57]. Consequently, pharmacological modulation of EndMT has emerged as a promising therapeutic strategy for limiting pathological vascular remodeling and improving the efficacy of anti-cancer treatments targeting the tumor vasculature [58].

Nevertheless, therapeutic inhibition of EndMT presents substantial challenges because this process may have both pathological and physiological functions. Complete suppression of endothelial plasticity could impair normal tissue repair and vascular adaptation. Future approaches may therefore need to focus on selectively targeting tumor-specific EndMT programs rather than globally blocking endothelial phenotypic transitions.

**Figure 2:**
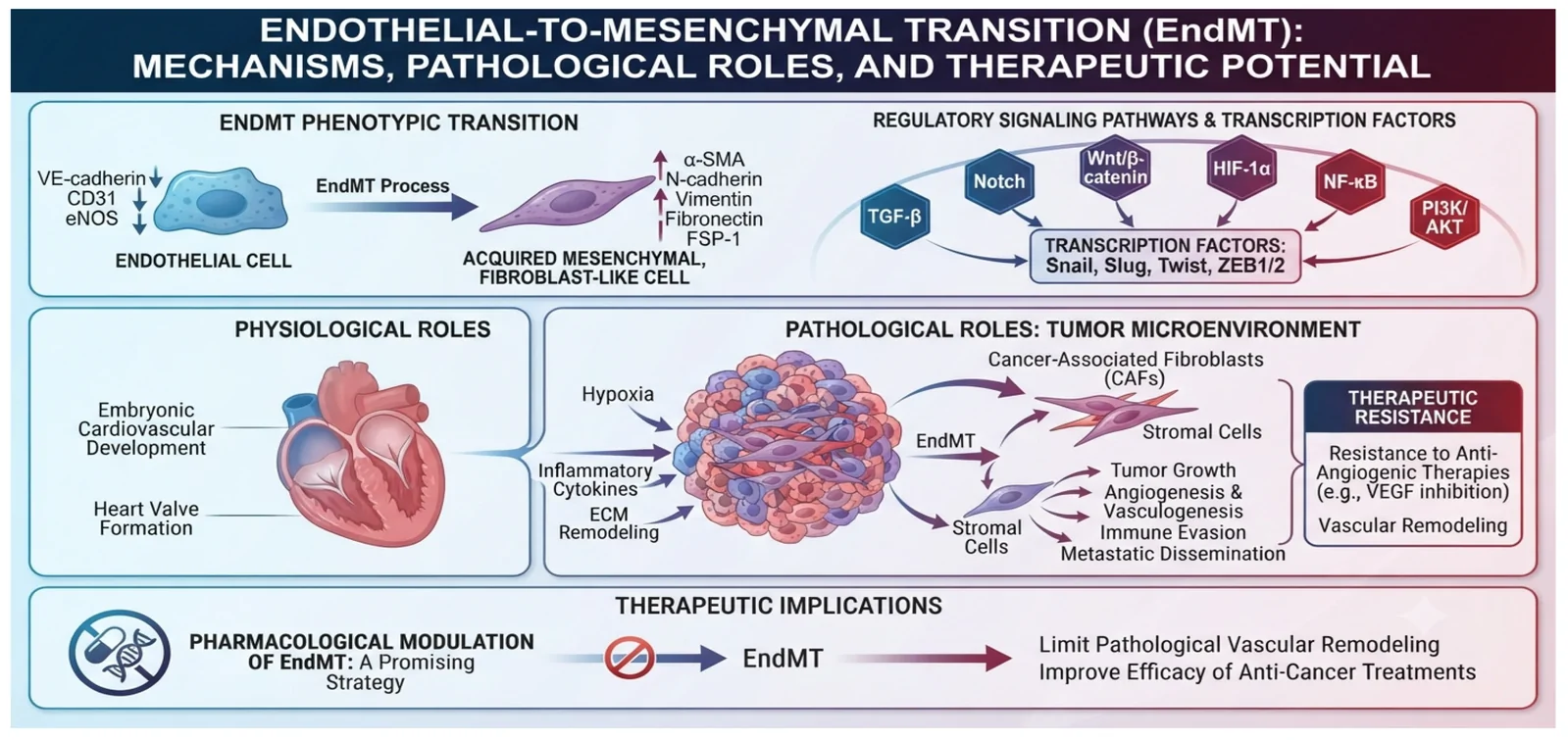
As illustrated in the figure, Endothelial-to-Mesenchymal Transition (EndMT) represents a dynamic phenotypic conversion where endothelial cells lose classic markers (such as VE-cadherin, CD31, and eNOS) and upregulate mesenchymal components (N-cadherin, vimentin, fibronectin, and FSP-1). This process is driven by key upstream signaling pathways—including TGF-beta, Notch, Wnt/beta-catenin, HIF-1$, NF-kappa B, and PI3K/AKT—which activate core transcription factors such as Snail, Slug, Twist, and ZEB1/2. While physiologically essential for embryonic cardiovascular development and heart valve formation, pathological EndMT is triggered within the tumor microenvironment by hypoxia, inflammatory cytokines, and extracellular matrix remodeling. This maladaptive transition generates cancer-associated fibroblasts (CAFs) and stromal cells that actively promote tumor growth, angiogenesis, vasculogenesis, immune evasion, and metastatic dissemination, while concurrently driving resistance to anti-angiogenic therapies like VEGF inhibition. Consequently, pharmacological modulation of EndMT emerges as a vital therapeutic strategy to limit pathological vascular remodeling and enhance the efficacy of precision cancer treatments.

See Figure 2 summarizing mechanisms of Endothelial-to-Mesenchymal Transition.

## 9. Mechanical Regulation and Mechanotransduction

Mechanical forces are fundamental regulators of vascular development and remodeling, influencing endothelial cell behavior throughout both physiological vasculogenesis and tumor-associated neovascularization. Mechanotransduction refers to the process by which endothelial cells detect and convert mechanical stimuli into intracellular biochemical signals that regulate gene expression, proliferation, migration, differentiation, and survival [59]. The principal mechanical stimuli include fluid shear stress generated by blood flow, cyclic stretch resulting from pulsatile vascular pressure, and extracellular matrix (ECM) stiffness, all of which influence vascular architecture and endothelial plasticity [60].

Endothelial cells sense these mechanical cues through mechanosensitive structures, including integrins, VE-cadherin, platelet endothelial cell adhesion molecule-1 (PECAM-1/CD31), focal adhesion kinase (FAK), ion channels, and the endothelial glycocalyx. Activation of these mechanoreceptors initiates signaling cascades involving phosphatidylinositol 3-kinase/protein kinase B (PI3K/AKT), mitogen-activated protein kinase (MAPK/ERK), RhoA/ROCK, Yes-associated protein/transcriptional co-activator with PDZ-binding motif (YAP/TAZ), and Krüppel-like factor 2 (KLF2), ultimately regulating endothelial phenotype and vascular remodeling [61].

Within the tumor microenvironment, increased matrix stiffness, elevated interstitial pressure, and abnormal blood flow produce sustained mechanotransduction signaling that promotes endothelial dysfunction, endothelial-to-mesenchymal transition (EndMT), vasculogenesis, and angiogenesis [62]. Mechanical activation of YAP/TAZ and focal adhesion pathways also enhances vascular remodeling, tumor cell invasion, and metastatic dissemination. Consequently, mechanotransduction has emerged as an important therapeutic target, with strategies aimed at normalizing tumor biomechanics and inhibiting mechanoresponsive signaling pathways, showing potential to improve vascular function and enhance the efficacy of anti-cancer therapies [63].

**Figure 3:**
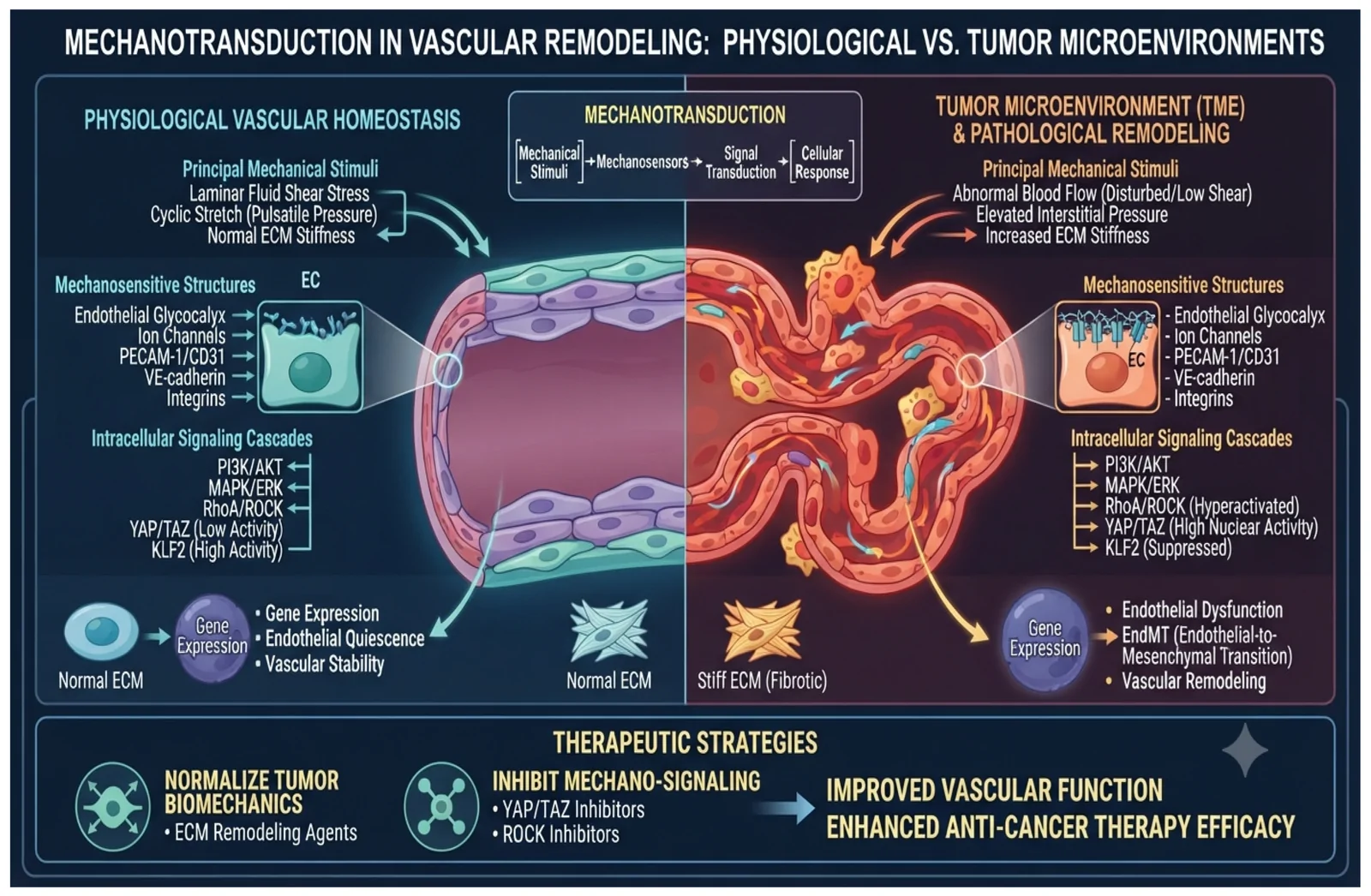
Endothelial mechanotransduction contrasts sharply between physiological vascular homeostasis and the tumor microenvironment. Under normal conditions, laminar fluid shear stress, cyclic stretch, and normal extracellular matrix (ECM) stiffness maintain endothelial quiescence and vascular stability via high KLF2 and low YAP/TAZ activity. Conversely, within the tumor microenvironment, abnormal blood flow, elevated interstitial pressure, and increased ECM stiffness hyperactivate mechanosensitive structures and intracellular signaling cascades (such as RhoA/ROCK and nuclear YAP/TAZ), driving endothelial dysfunction, endothelial-to-mesenchymal transition (EndMT), and pathological vascular remodeling. Targeting these biomechanical pathways using ECM-normalizing or mechano-signaling inhibitors (such as YAP/TAZ or ROCK inhibitors) offers a promising strategy to improve vascular function and enhance anti-cancer therapy efficacy.

Importantly, whether biomechanical normalization can be effectively translated into clinical practice remains uncertain. While reducing extracellular matrix stiffness and abnormal vascular forces may improve drug delivery, excessive vascular normalization could potentially alter tumor evolution and immune interactions in unpredictable ways. Thus, future research should determine the optimal balance between modifying tumor mechanics and preserving beneficial vascular functions. See Figure 3 which summarizes the effects of mechanical forces involved in vascular remodeling.

## 10. Non-Coding RNAs Regulating Tumor Vasculogenesis

Non-coding RNAs (ncRNAs) have emerged as critical epigenetic regulators of tumor vasculogenesis by modulating the expression of genes involved in endothelial progenitor cell (EPC) mobilization, endothelial differentiation, vascular remodeling, and communication within the tumor microenvironment. The major classes include microRNAs (miRNAs), long non-coding RNAs (lncRNAs), and circular RNAs (circRNAs), which regulate gene expression through transcriptional, post-transcriptional, and translational mechanisms [64]. Dysregulation of these molecules contributes to pathological vascular development and has been implicated in numerous human malignancies.

Among the best-characterized regulators, **miR-126** promotes endothelial survival and vascular integrity by enhancing VEGF signaling, whereas **miR-21**, **miR-210**, and **miR-155** are frequently overexpressed in hypoxic tumors and stimulate vasculogenesis through activation of HIF-1α, PI3K/AKT, and inflammatory signaling pathways [65]. Conversely, anti-angiogenic microRNAs, including **miR-15a** and **miR-16**, suppress endothelial proliferation by inhibiting VEGF expression. Long non-coding RNAs such as **MALAT1**, **HOTAIR**, and **H19** further regulate endothelial cell proliferation, migration, and endothelial-to-mesenchymal transition (EndMT), while several circular RNAs function as competing endogenous RNAs by sequestering vasculogenesis-related microRNAs [66].

From a clinical perspective, ncRNA signature represents emerging but not yet fully validated biomarker of tumor vascular remodeling. Circulating microRNAs, long non-coding RNAs, circular RNAs, and extracellular vesicle-associated RNA profiles have demonstrated prognostic and predictive potential in several malignancies, but their clinical implementation remains limited by variability in detection platforms, lack of standardized thresholds, and tumor-specific differences. Future studies integrating liquid biopsy approaches, spatial transcriptomics, and multi-omics analyses may facilitate the development of clinically applicable biomarkers reflecting tumor vasculogenic activity and treatment response.

In addition, tumor-derived extracellular vesicles and exosomes transport ncRNAs between cancer cells, endothelial cells, stromal cells, and EPCs, thereby remodeling the tumor microenvironment and facilitating pathological neovascularization [67]. Given their stability, tissue specificity, and regulatory capacity, ncRNAs represent promising biomarkers of tumor progression and attractive therapeutic targets. Strategies aimed at modulating oncogenic ncRNAs or restoring tumor-suppressive ncRNAs may complement conventional anti-angiogenic therapies and improve the management of vasculogenesis-driven malignancies [68].

## 11. Anti-Vasculogenic Therapeutic Strategies

Targeting tumor vasculogenesis represents an emerging therapeutic concept that may complement established anti-angiogenic approaches; however, clinical validation remains limited. While anti-angiogenic therapies targeting VEGF/VEGFR signaling have demonstrated clinical efficacy in multiple malignancies, most anti-vasculogenic strategies remain at the preclinical or early translational stage, and their therapeutic benefit in patients has not yet been definitively established. While anti-angiogenic agents primarily inhibit endothelial sprouting from pre-existing blood vessels, anti-vasculogenic strategies aim to prevent the recruitment, mobilization, differentiation, and functional integration of endothelial progenitor cells (EPCs) into the tumor vasculature. Because tumors frequently activate alternative vascularization mechanisms following inhibition of angiogenesis, simultaneous targeting of both processes may provide more durable therapeutic responses [69].

The **vascular endothelial growth factor (VEGF)/VEGF receptor (VEGFR)** signaling pathway remains the principal therapeutic target in tumor vascularization.

Among vascular-targeted therapies, VEGF pathway inhibition represents the most clinically validated strategy. Bevacizumab and VEGFR tyrosine kinase inhibitors have demonstrated therapeutic efficacy and received regulatory approval for several cancers, including colorectal cancer, renal cell carcinoma, hepatocellular carcinoma, and other malignancies. However, these agents primarily target angiogenic signaling rather than specifically inhibiting vasculogenesis, and treatment resistance frequently develops through adaptive vascular remodeling mechanisms.

Monoclonal antibodies such as **bevacizumab** and VEGFR-targeting tyrosine kinase inhibitors, including **sunitinib**, **sorafenib**, **pazopanib**, **axitinib**, and **lenvatinib**, inhibit endothelial proliferation, vascular permeability, and tumor blood vessel formation [70]. However, clinical studies have demonstrated that VEGF inhibition alone rarely produces sustained responses because hypoxia induced by therapy often activates compensatory vasculogenic pathways mediated by endothelial progenitor cell recruitment.

Consequently, increasing attention has focused on disrupting the **HIF-1α/VEGF/SDF-1(CXCL12)/CXCR4 signaling axis**, which regulates EPC mobilization from the bone marrow and homing to hypoxic tumor regions. Pharmacological inhibition of CXCR4 using agents such as **plerixafor** has shown the potential to reduce EPC recruitment and suppress tumor vasculogenesis in preclinical models.

Importantly, these findings are currently supported mainly by experimental studies, and the clinical effectiveness of CXCR4 inhibition as a direct anti-vasculogenic strategy remains under investigation. Although CXCR4-targeting approaches have demonstrated biological activity in several disease contexts, their specific contribution to controlling tumor vascularization requires further clinical validation.

Likewise, inhibition of HIF-1α may simultaneously reduce the expression of multiple pro-vasculogenic mediators, including VEGF, SDF-1, angiopoietins, and platelet-derived growth factor (PDGF), thereby limiting vascular remodeling and metastatic progression [71].

Additional therapeutic strategies target developmental signaling pathways that regulate endothelial differentiation and vascular maturation. Inhibitors of **Notch**, **DLL4**, **TGF-β**, **PI3K/AKT**, and **Wnt/β-catenin** signaling have demonstrated anti-vasculogenic effects by disrupting endothelial plasticity, vascular remodeling, and endothelial-to-mesenchymal transition (EndMT) [72].

Nevertheless, evidence supporting these approaches is predominantly derived from preclinical cancer models. For example, DLL4/Notch inhibition can disrupt tumor vascular development experimentally, but clinical translation has been complicated by toxicity concerns and the complex role of Notch signaling in normal vascular homeostasis. Similarly, targeting Wnt, TGF-β, and PI3K/AKT pathways requires careful consideration because these pathways regulate multiple physiological processes beyond tumor vasculogenesis.

However, the translation of secondary vasculogenic pathway inhibitors into clinical practice has been limited by their involvement in essential physiological processes. CXCR4 signaling plays critical roles in hematopoietic stem cell trafficking, immune cell migration, and tissue repair; therefore, systemic CXCR4 inhibition may interfere with normal regenerative responses and immune homeostasis. Similarly, Notch/DLL4 signaling is required for normal endothelial differentiation, vessel maturation, and maintenance of vascular integrity. Clinical attempts to inhibit DLL4 have been associated with dose-limiting toxicities, including vascular abnormalities and hepatic complications, reflecting the challenge of selectively targeting tumor-associated vascular plasticity while preserving normal vascular function. In addition, pathways such as TGF-β, Wnt, and PI3K/AKT regulate diverse cellular functions, including fibrosis control, metabolism, immune regulation, and tissue regeneration, making systemic inhibition potentially harmful. These limitations highlight the necessity of developing tumor-specific delivery systems, predictive biomarkers, and context-dependent therapeutic strategies to maximize anti-vasculogenic efficacy while minimizing toxicity.

Combination therapies simultaneously targeting multiple pathways may reduce compensatory signaling and improve treatment efficacy.

Emerging approaches also focus on **non-coding RNAs (ncRNAs)**, extracellular vesicles, and mechanotransduction pathways.

These approaches remain largely experimental, with current evidence primarily originating from cellular and animal studies. Although modulation of ncRNAs, YAP/TAZ, and mechanotransduction pathways represents an attractive therapeutic concept, their clinical applicability is limited by challenges related to delivery, specificity, and potential effects on normal tissue repair mechanisms.

Table 3 summarizes the currently investigated anti-vasculogenic strategies in cancer and their corresponding evidence levels.

Therapeutic modulation of oncogenic microRNAs, long non-coding RNAs, or circular RNAs may suppress endothelial progenitor cell function and tumor vascular remodeling, whereas inhibition of YAP/TAZ and focal adhesion kinase (FAK) signaling may normalize abnormal tumor biomechanics and impair pathological vessel formation [73]. Furthermore, immunotherapy may indirectly inhibit vasculogenesis by reducing the production of inflammatory cytokines and pro-vasculogenic mediators within the tumor microenvironment. See Table 4 which summarizes the main anti-vasculogenic strategies in cancer.

Despite promising experimental findings, major challenges limit the clinical translation of anti-vasculogenic therapies. Tumor heterogeneity, pathway redundancy, adaptive resistance, and the absence of validated biomarkers remain significant barriers. Currently, VEGF/VEGFR-directed therapies represent the only extensively clinically established vascular-targeted strategies, whereas most approaches specifically targeting vasculogenesis remain investigational. Future clinical studies should focus on identifying patients with vasculogenesis-dependent tumors and evaluating rational combinations of anti-vasculogenic strategies with anti-angiogenic agents, immunotherapy, chemotherapy, or molecularly targeted treatments. A more comprehensive understanding of the molecular mechanisms governing tumor vasculogenesis will facilitate the development of precision oncology approaches capable of improving long-term clinical outcomes while minimizing treatment resistance [74].

## 12. Current Limitations, Conflicting Evidence, and Future Perspectives in Tumor Vasculogenesis

Despite substantial advances in understanding tumor vasculogenesis, several challenges limit the successful translation of anti-vasculogenic strategies into clinical practice. One major obstacle is the considerable heterogeneity among tumor types, in which the contribution of endothelial progenitor cells (EPCs), vasculogenic pathways, and vascular remodeling mechanisms varies according to cancer origin, molecular subtype, disease stage, and microenvironmental conditions [75]. Furthermore, the functional definition and identification of EPCs remain controversial due to the lack of universally accepted specific markers, complicating the accurate evaluation of their contribution to tumor vascularization [76].

A major unresolved issue in the field is the extent to which vasculogenesis contributes directly to functional tumor blood vessel formation in humans. Although several experimental studies have demonstrated incorporation of bone marrow-derived or progenitor-like cells into tumor vasculature, other investigations using lineage-tracing approaches have suggested that their direct contribution may be considerably lower than initially proposed. These discrepancies may result from differences in experimental models, tumor types, EPC identification strategies, and methodological approaches used to distinguish true endothelial differentiation from paracrine support of angiogenesis.

Furthermore, distinguishing vasculogenesis from other vascular adaptation mechanisms remains challenging. Tumors frequently utilize multiple overlapping processes, including angiogenesis, vessel co-option, vascular mimicry, and endothelial plasticity, making it difficult to define the relative contribution of individual mechanisms. The majority of current knowledge originates from preclinical models, which may not fully reflect the complex interactions between cancer cells, immune populations, stromal components, extracellular matrix remodeling, and metabolic gradients present in human tumors.

Another important challenge is the extensive redundancy and adaptability of vascular signaling networks. Inhibition of a single pathway, such as VEGF signaling, frequently results in activation of compensatory mechanisms involving HIF-1α, CXCL12/CXCR4, Notch, TGF-β, or alternative vascularization processes, leading to therapeutic resistance [77]. Additionally, systemic inhibition of vascular pathways may affect physiological vascular repair and normal tissue homeostasis, emphasizing the need for selective tumor-targeted approaches.

Future research should focus on identifying reliable biomarkers capable of predicting vasculogenic activity and therapeutic response. Integration of single-cell sequencing, spatial transcriptomics, advanced imaging technologies, and artificial intelligence-based analysis may provide deeper insights into tumor vascular heterogeneity. Combination therapies targeting vasculogenesis together with anti-angiogenic agents, immunotherapy, and molecularly targeted treatments represent promising strategies. Ultimately, a more precise understanding of vascular plasticity and tumor-specific vasculogenic mechanisms will facilitate the development of personalized therapies aimed at improving cancer outcomes.

## 13. Conclusions

Tumor vasculogenesis has emerged as an important component of cancer vascular adaptation that extends beyond classical angiogenesis and contributes to tumor growth, metastatic progression, and resistance to vascular-targeted therapies. Major advances in this field have revealed that vasculogenic processes are regulated by complex interactions among endothelial progenitor cells, hypoxia-responsive pathways, developmental signaling networks (including VEGF, Notch, Wnt, BMP, TGF-β, PI3K/AKT, and HIF-1α), and tumor microenvironmental factors such as extracellular matrix remodeling, inflammation, and mechanical cues. These findings have expanded the understanding of tumor vascularization from a predominantly angiogenesis-centered model toward a broader concept of vascular plasticity.

Despite these advances, several critical issues remain unresolved. The quantitative contribution of vasculogenesis to human tumor vascular formation remains controversial, partly due to inconsistent definitions of endothelial progenitor cells and limitations in distinguishing vasculogenesis from angiogenesis, vascular mimicry, and endothelial plasticity. Furthermore, tumor heterogeneity, signaling pathway redundancy, and the absence of reliable predictive biomarkers continue to limit successful clinical translation.

From a therapeutic perspective, targeting tumor vasculogenesis represents a promising complementary strategy rather than a replacement for established anti-angiogenic approaches. Future clinical applications will likely require patient-specific approaches combining anti-vasculogenic therapies with anti-angiogenic agents, immunotherapy, and molecularly targeted treatments. Integration of single-cell sequencing, spatial profiling, and advanced imaging approaches may enable identification of patients most likely to benefit from vascular-targeted interventions and facilitate the development of precision therapies aimed at overcoming tumor vascular adaptation and treatment resistance.

## Figures and Tables

**Table 1:** Molecular and Cellular Mechanisms Regulating Physiological Vasculogenesis During Embryonic Development.

Mechanism/Process	Main Molecular Components	Cellular Effects	Biological Significance in Vasculogenesis
Mesoderm specification and hemangioblast formation	Mesoderm-derived progenitor cells, hemangioblasts, FGF, BMP signaling	Induction of bipotent progenitor cells capable of endothelial and hematopoietic differentiation	Establishes the cellular origin of the first vascular structures during embryogenesis
Endothelial lineage commitment	VEGF-A, VEGFR-2 (KDR/Flk-1), ETV2, SCL/TAL1, GATA2, ERG	Activation of endothelial-specific gene expression and differentiation of progenitor cells into endothelial cells	Determines endothelial cell identity and initiates primitive vessel formation
VEGF-mediated endothelial activation	VEGF-A/VEGFR-2 signaling, PI3K/AKT, MAPK/ERK, PLCγ pathways	Promotes endothelial cell survival, proliferation, migration, and differentiation	Acts as the central signaling axis driving endothelial development and vascular network formation
Endothelial cell migration and vascular cord assembly	VE-cadherin, integrins, fibronectin, laminin, extracellular matrix interactions	Coordinated endothelial migration, cell adhesion, and organization into primitive vascular cords	Enables formation of the primary vascular plexus
Lumen formation and endothelial polarization	Intracellular vacuole formation, cytoskeletal remodeling, endothelial polarity pathways	Fusion of intracellular vacuoles, establishment of vascular lumen, endothelial organization	Converts solid endothelial cords into functional vascular channels
Extracellular matrix remodeling and stabilization	Fibronectin, laminin, endothelial extracellular matrix secretion	Provides structural support and regulates endothelial adhesion and migration	Maintains integrity and organization of developing vessels
Arterial–venous specification	Notch signaling, DLL4, Notch receptors, EphrinB2, EphB4	Differentiation of endothelial cells into arterial or venous phenotypes	Establishes vascular identity and functional specialization of vessels
Pericyte and vascular smooth muscle recruitment	PDGF-B, TGF-β signaling	Recruitment of mural cells and enhancement of vascular stability	Promotes vessel maturation and prevents vascular regression
Mechanical regulation and mechanotransduction	Shear stress, KLF2, YAP/TAZ, endothelial nitric oxide synthase (eNOS)	Regulation of endothelial differentiation, vascular remodeling, and response to blood flow	Integrates biomechanical forces with molecular vascular development
Endothelial–microenvironment communication	Growth factors, extracellular matrix signals, paracrine interactions	Coordination between endothelial cells and surrounding stromal components	Ensures adaptive vascular growth and maturation
Vascular remodeling after initial plexus formation	VEGF, Notch, TGF-β, angiopoietins, matrix interactions	Pruning, stabilization, and restructuring of primitive vessels	Generates a mature and functional circulatory network

**Table 2:** Comparison of Tumor Vasculogenesis and Angiogenesis.

Feature	Tumor Vasculogenesis	Tumor Angiogenesis
Definition	De novo formation of blood vessels through recruitment and differentiation of endothelial progenitor cells (EPCs).	Formation of new blood vessels by sprouting, branching, and remodeling of pre-existing vessels.
Primary cellular source	Bone marrow-derived endothelial progenitor cells (EPCs) and other circulating vascular progenitors.	Resident mature endothelial cells from existing vasculature.
Physiological role	Predominant mechanism during embryonic vascular development; limited role in adult vascular repair.	Major mechanism of physiological vascular growth, wound healing, and tissue regeneration in adults.
Role in cancer	Promotes tumor vascularization through EPC recruitment and incorporation into newly formed vessels.	Expands the existing vascular network to meet the metabolic demands of growing tumors.
Main initiating stimulus	Tumor hypoxia and ischemia leading to progenitor cell mobilization.	Local tissue hypoxia and secretion of proangiogenic growth factors.
Principal molecular mediators	HIF-1α, VEGF, SDF-1/CXCL12, CXCR4, angiopoietins, nitric oxide.	VEGF, FGF, PDGF, angiopoietins, Notch, integrins.
Mechanism of vessel formation	Recruitment, migration, proliferation, and differentiation of EPCs into endothelial cells that form new vascular structures.	Activation, proliferation, migration, and sprouting of mature endothelial cells from existing vessels.
Contribution of EPCs	Essential; EPCs directly participate in vessel formation and secrete pro-vasculogenic factors.	Minimal; mature endothelial cells are the principal contributors.
Tumor microenvironment	Strongly influenced by hypoxia, inflammatory cytokines, chemokines, and bone marrow-derived cells.	Predominantly regulated by local endothelial activation and extracellular matrix remodeling.
Major biological functions	Supports tumor growth, vascular remodeling, metastasis, recurrence, and therapeutic resistance.	Provides oxygen and nutrient supply necessary for continued tumor expansion.
Clinical significance	Important mechanism of tumor revascularization following anti-angiogenic therapy; potential therapeutic target.	Established therapeutic target with approved anti-VEGF and anti-angiogenic agents.
Representative therapeutic targets	EPC mobilization, HIF-1α, SDF-1/CXCR4 axis, VEGF signaling, bone marrow-derived vascular progenitors.	VEGF/VEGFR, FGFs, PDGF, angiopoietins, Notch signaling, endothelial proliferation pathways.

**Table 3:** Antivasculotropic agents and their clinical/preclinical evidence.

Strategy	Target	Evidence Level
Bevacizumab/VEGFR inhibitors	VEGF pathway	Clinical evidence
CXCR4 inhibition	EPC recruitment	Preclinical/early translational
HIF-1α inhibition	Hypoxia response	Experimental
DLL4/Notch inhibition	Vascular differentiation	Preclinical
Wnt/TGF-β/PI3K inhibitors	Vascular plasticity	Preclinical
YAP/TAZ/FAK inhibition	Mechanotransduction	Experimental

**Table 4:** Anti-Vasculogenic Therapeutic Strategies in Cancer.

Therapeutic Strategy	Primary Molecular Target(s)	Mechanism of Action	Representative Agents	Potential Clinical Benefit
VEGF/VEGFR inhibition	VEGF-A, VEGFR-1, VEGFR-2	Inhibits endothelial cell proliferation, migration, vascular permeability, and new vessel formation	Bevacizumab, Sunitinib, Sorafenib, Pazopanib, Axitinib, Lenvatinib	Reduces tumor vascularization and suppresses tumor growth
HIF-1α inhibition	HIF-1α	Suppresses hypoxia-induced transcription of VEGF, SDF-1, PDGF, and angiopoietins	HIF-1α inhibitors (investigational agents)	Limits hypoxia-driven vasculogenesis and tumor adaptation
CXCL12/CXCR4 blockade	SDF-1 (CXCL12), CXCR4	Prevents mobilization and homing of endothelial progenitor cells (EPCs) from bone marrow to tumors	Plerixafor	Inhibits EPC recruitment and pathological vasculogenesis
PI3K/AKT pathway inhibition	PI3K, AKT, mTOR	Reduces endothelial cell survival, proliferation, migration, and VEGF signaling	PI3K, AKT, and mTOR inhibitors	Suppresses vascular remodeling and tumor progression
Notch/DLL4 inhibition	Notch receptors, DLL4	Disrupts endothelial differentiation, arterial specification, and vascular maturation	Anti-DLL4 antibodies, γ-secretase inhibitors	Produces abnormal non-functional vessels and inhibits tumor perfusion
TGF-β pathway inhibition	TGF-β receptors, SMAD proteins	Suppresses EndMT, extracellular matrix remodeling, and vascular stabilization	TGF-β receptor inhibitors	Reduces vascular remodeling, fibrosis, and tumor invasion
Wnt/β-catenin inhibition	Wnt ligands, Frizzled receptors, β-catenin	Inhibits endothelial differentiation and vascular progenitor maintenance	Wnt pathway inhibitors	Restrains vasculogenesis and tumor cell proliferation
Non-coding RNA-based therapy	miRNAs, lncRNAs, circRNAs	Modulates expression of vasculogenesis-related genes and signaling pathways	miRNA mimics, antagomiRs, lncRNA-targeting oligonucleotides	Regulates endothelial plasticity and tumor vascular remodeling
Mechanotransduction inhibition	YAP/TAZ, FAK, Integrins	Blocks mechanosensitive signaling induced by matrix stiffness and abnormal blood flow	FAK inhibitors, YAP/TAZ inhibitors	Normalizes tumor vasculature and reduces invasion and metastasis
Immunomodulatory strategies	Immune cells, inflammatory cytokines	Decreases inflammatory mediators that stimulate EPC recruitment and vascular remodeling	Immune checkpoint inhibitors (combination strategies)	Indirect inhibition of tumor vasculogenesis and enhancement of anti-tumor immunity
Combination therapies	Multiple signaling pathways	Simultaneous inhibition of angiogenesis, vasculogenesis, and tumor immune escape	Anti-VEGF + Immunotherapy; Anti-VEGF + TKIs; Anti-VEGF + CXCR4 inhibitors	Overcomes therapeutic resistance and improves long-term clinical outcomes

Abbreviations: VEGF, vascular endothelial growth factor; VEGFR, vascular endothelial growth factor receptor; HIF-1α, hypoxia-inducible factor-1α; SDF-1/CXCL12, stromal-derived factor-1; CXCR4, C-X-C chemokine receptor 4; PI3K, phosphatidylinositol 3-kinase; AKT, protein kinase B; mTOR, mammalian target of rapamycin; DLL4, Delta-like ligand 4; TGF-β, transforming growth factor-β; EndMT, endothelial-to-mesenchymal transition; FAK, focal adhesion kinase; EPCs, endothelial progenitor cells.

## Data Availability

All data supporting the findings of this review are derived from previously published studies that are publicly available. The articles analyzed and discussed in this manuscript are accessible through the corresponding publications listed in the reference list.

## Abbreviations

AKT	Protein kinase B
AIF	Apoptosis-inducing factor
AUC	Area under the curve
BMP	Bone morphogenetic protein
CD31	Cluster of differentiation 31 (platelet endothelial cell adhesion molecule-1)
CXCL12	C-X-C motif chemokine ligand 12 (stromal-derived factor-1, SDF-1)
CXCR4	C-X-C chemokine receptor type 4
DLL4	Delta-like ligand 4
ECM	Extracellular matrix
eNOS	Endothelial nitric oxide synthase
EPCs	Endothelial progenitor cells
ERG	ETS-related gene
EndMT	Endothelial-to-mesenchymal transition
FGF	Fibroblast growth factor
FAK	Focal adhesion kinase
G-CSF	Granulocyte colony-stimulating factor
HIF-1α	Hypoxia-inducible factor-1 alpha
HPV	Human papillomavirus
KDR	Kinase insert domain receptor (VEGFR-2)
KLF2	Krüppel-like factor 2
lncRNAs	Long non-coding RNAs
MAPK	Mitogen-activated protein kinase
miRNAs	MicroRNAs
mTOR	Mammalian target of rapamycin
ncRNAs	Non-coding RNAs
NF-κB	Nuclear factor kappa B
NO	Nitric oxide
PDGF	Platelet-derived growth factor
PECAM-1	Platelet endothelial cell adhesion molecule-1 (CD31)
PI3K	Phosphatidylinositol 3-kinase
PLCγ	Phospholipase C gamma
SCL/TAL1	Stem cell leukemia/T-cell acute lymphocytic leukemia 1 transcription factor
SDF-1	Stromal-derived factor-1 (CXCL12)
SMAD	Mothers against decapentaplegic homolog proteins (TGF-β signaling mediators)
TGF-β	Transforming growth factor beta
VE-cadherin	Vascular endothelial cadherin
VEGF	Vascular endothelial growth factor
VEGFR	Vascular endothelial growth factor receptor
VHL	Von Hippel–Lindau tumor suppressor protein
Wnt	Wingless-related integration site signaling pathway
YAP/TAZ	Yes-associated protein/transcriptional co-activator with PDZ-binding motif
TKIs	Tyrosine kinase inhibitors
CXCR	C-X-C chemokine receptor
PD-L1	Programmed death-ligand 1
CTLA-4	Cytotoxic T-lymphocyte-associated protein 4
